# First Case Report of Bilateral Avascular Necrosis in an Eosinophilic Esophagitis Patient on Swallowed Fluticasone

**DOI:** 10.7759/cureus.72815

**Published:** 2024-11-01

**Authors:** Eyad Alakrad, Khalifa Al Teneiji, Shaima W Khan, Mohamed Al zaabi, Najla Saleh Ben Ghashir, Maryam Alahmad

**Affiliations:** 1 Gastroenterology, Sheikh Shakhbout Medical City, Abu Dhabi, ARE; 2 Gastroenterology, Khalifa University, Abu Dhabi, ARE; 3 Gastroenterology and Hepatology, Sheikh Shakhbout Medical City, Abu Dhabi, ARE; 4 Pathology, Sheikh Shakhbout Medical City, Abu Dhabi, ARE

**Keywords:** avascular necrosis, dupilumab, eosinophilic esophagitis, medication adverse effects, swallowed fluticasone

## Abstract

Avascular necrosis (AVN) of the femoral head is a rare complication that can be associated with prolonged use of glucocorticoids. On reviewing the literature, this is the first case of bilateral AVN secondary to the long use of topical steroids in an eosinophilic esophagitis (EoE) patient. A 28-year-old man presented to our gastroenterology clinic for worsening fibrostenotic (EoE) despite being on swallowed fluticasone administered through an inhaler for seven years. The latter was stopped, and the patient was commenced on dupilumab. The patient was referred to orthopedics to manage his limping and groin pain. Bilateral hip replacements were performed when bilateral AVN was confirmed on MRI of both hips. The patient’s EoE symptoms resolved after endoscopic dilation and an ongoing course of dupilumab. Furthermore, dupilumab showed an excellent control of the disease activity based on esophageal biopsies. To our knowledge, this is the first case report of such an entity. Our case highlights the need to promote awareness of such serious side effects of prolonged use of topical corticosteroids among healthcare providers, even if administered through a metered inhaler.

## Introduction

Eosinophilic esophagitis (EoE) is a chronic immune-mediated, food-driven Th2 inflammatory disorder that affects children and adults. EoE prevalence has doubled over the past two decades, affecting one in 1,500-2000 persons [1]. The exact pathophysiology of EoE is not well known, but it is thought to be secondary to immunologic reactions with the interplay of genetic, environmental, and host defense factors. Exposure to aeroallergens, food, and environmental antigens induces a cascade of allergic and immunologic reactions, which lead to inflammation and remodeling of esophageal mucosa [2].

The most common clinical presentations in adults are dysphagia to solid food, chest pain, and heartburn. History of various atopic disorders may be present as well.

Diagnosis is usually made by the presence of esophageal dysfunction symptoms, 15 or more eosinophils per high-power field (eos/hpf) on esophageal biopsy, and exclusion of other disorders that can cause esophageal eosinophilia [3]. Despite the lack of general consensus, standard-of-care treatments include oral proton pump inhibitor (PPI), swallowed topical steroids, six-food elimination diet (SFED), monoclonal antibody dupilumab, and, in case of strictures, esophageal dilatation [4].

Avascular necrosis (AVN) of the femoral head is a well-recognized complication of oral and topical steroid therapy [5]. Moreover, inhaled steroids were also accused of causing aseptic necrosis to the patella and femoral heads, according to multiple reports [6]. However, AVN of the femoral head secondary to the swallowed steroid therapy is rare. Here, we describe the first case of bilateral AVN of femoral heads after the swallowed topical steroid therapy.

Our case was presented as a poster presentation at the United European Gastroenterology (UEG) Week in 2024.

## Case presentation

A 28-year-old Emirati male presented with refractory EoE to our gastroenterology department, motility clinic at Sheikh Shakhbout Medical City (SSMC), Abu Dhabi, in July 2023. His symptoms were mainly dysphagia with severe regurgitation with excessive chewing and tolerating only small bites. Surprisingly, there is no history of any atopy or allergies. Although the patient has had these symptoms since the age of 10, the diagnosis of EoE was not made until he sought a second opinion abroad in 2011 at 16 years old. At that time, an esophagogastroduodenoscopy (EGD) revealed a stricture at the gastroesophageal junction (GEJ), which required balloon dilation, and endoscopic changes consistent with EoE were biopsied. The esophageal biopsies showed >15 eos/hpf and few eosinophilic micro-abscesses. The patient was started then on PPI and lost follow-up until November 2016, when he presented to the same center abroad with worsening symptoms. His second EGD then showed a worsening stricture at the GEJ, which required controlled radial expansion (CRE) balloon dilation at 12 mm. He was managed at this point with oral esomeprazole 40 mg twice a day and tapering doses of oral prednisolone 20 mg twice a day for less than a month. A follow-up EGD within a month reported moderate esophagitis with improving stricture in December 2016. The correlating pathology report mentioned the presence of mild eosinophilic micro-abscesses in the esophagus.

Since the patient was still symptomatic, the decision was made by his physician then to establish him on swallowed fluticasone from a multi-dose inhaler (MDI) along with his maintenance dose of esomeprazole 40 mg twice a day.

Between the years 2016 and 2023, the patient was maintained on the same combination of swallowed fluticasone and high doses of different PPIs, including but not limited to pantoprazole, omeprazole, and rabeprazole. During this period, he did undergo a few EGD with esophageal balloon dilations at the same gastroenterology center where his diagnosis was established. The limited endoscopy reports mentioned improving ring furrows but not complete resolution, and only a little information about the esophageal biopsy results was available to us. The patient was reporting only partial improvement, and he learned how to live with his symptoms as he described them. He claims no food-elimination diet was offered to him then. 

In March 2023, the patient developed bilateral AVN of the head of the femurs, which required surgical correction by bilateral core decompression of the femoral heads with Arthrex expandable reamer. Subsequently, he had a bilateral femoral replacement done in stages in November 2023 by an orthopedic team in another facility in UAE. 

**Figure 1:**
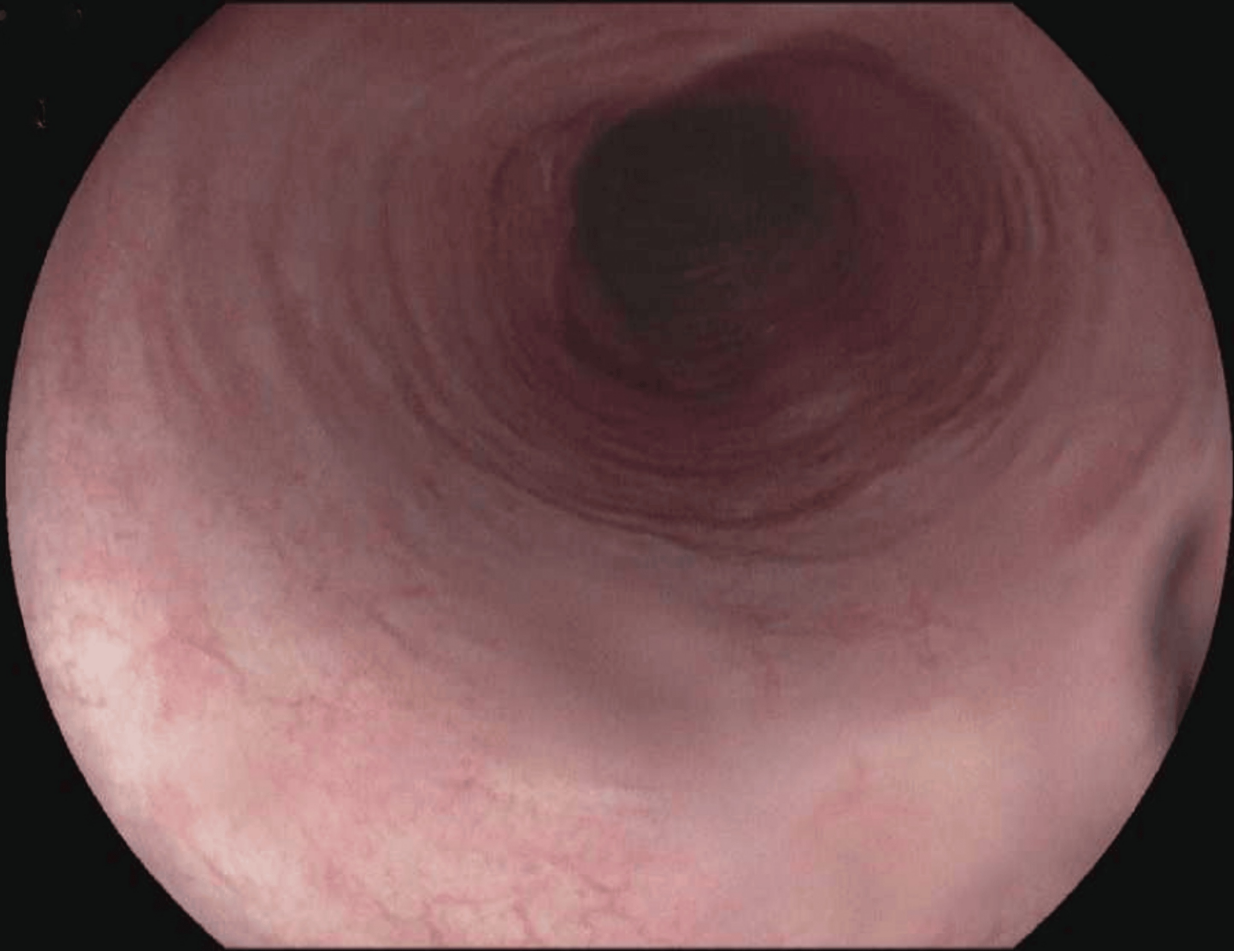
EGD showing mucosal changes consistent with EoE EGD, esophagogastroduodenoscopy; EoE, eosinophilic esophagitis

Our center assumed the patient’s care in July 2023. On his first EGD with us, his EoE endoscopic reference score (EoE-EREFS) was 4; edema: 1, rings: 1, exudates: 0, furrows: 1, and stricture: 1. His esophageal biopsies demonstrated 10 eos/hpf with basal cell hyperplasia in the distal esophagus and 0 eos/hpf with no significant histological changes in the proximal esophagus (Figure 1). After an elaborate discussion with the patient about his future management, he agreed to stop fluticasone, initiate dupilumab 300 mg subcutaneous injection weekly, and establish care with an allergist/immunologist. He declined to consider any food elimination diet; however, pantoprazole 40 mg twice a day was continued because erosive gastritis was found during endoscopy in addition to his typical symptoms of heartburn and regurgitation in the view of the GEJ stricture, which was not clear if it was related to the EoE or GERD disease activity. The patient was also agreeable to consider future endoscopic evaluations with or without dilation to direct his therapy and management as indicated.

**Figure 2:**
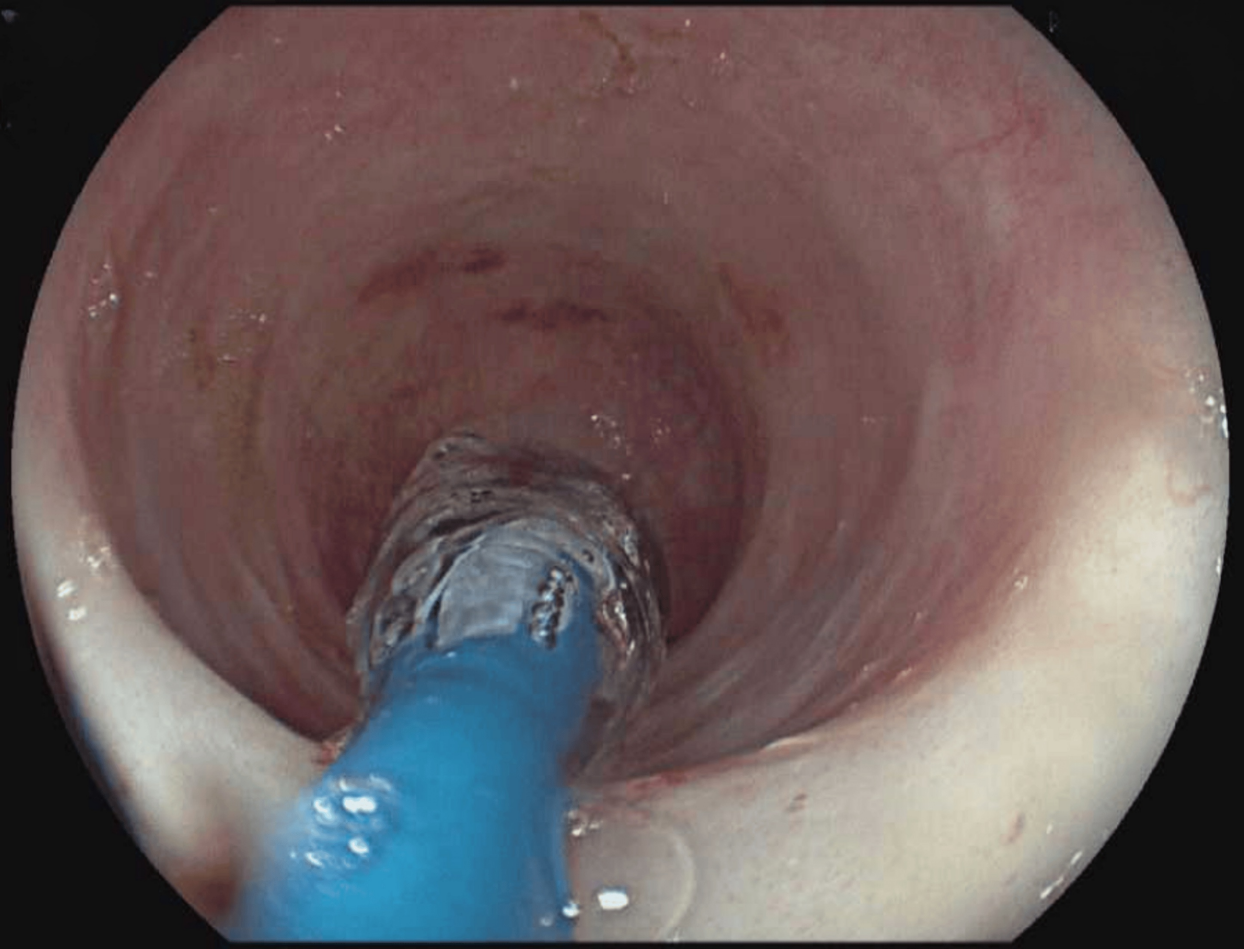
EGD predilation EGD, esophagogastroduodenoscopy

**Figure 3:**
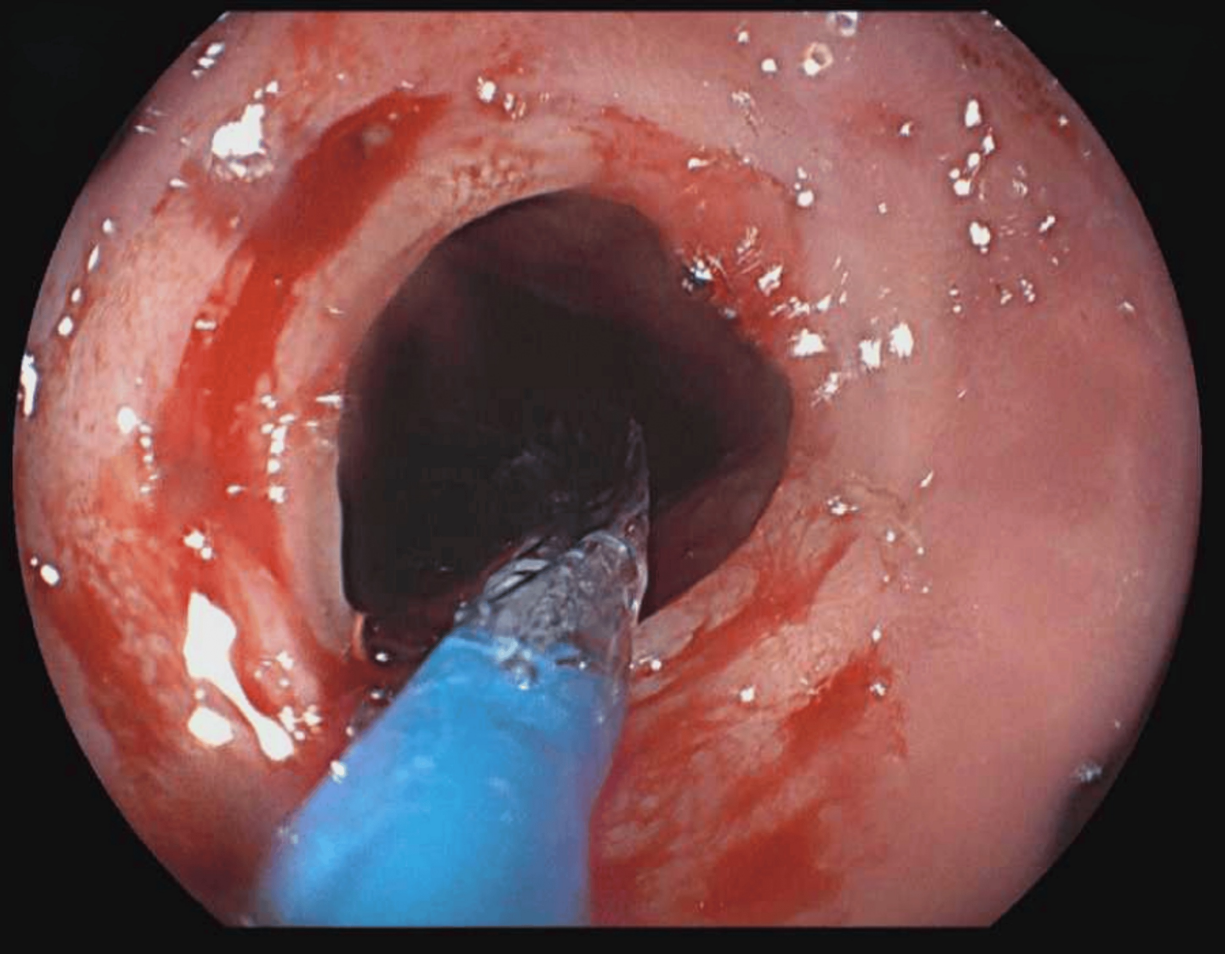
EGD postdilation EGD, esophagogastroduodenoscopy

**Figure 4:**
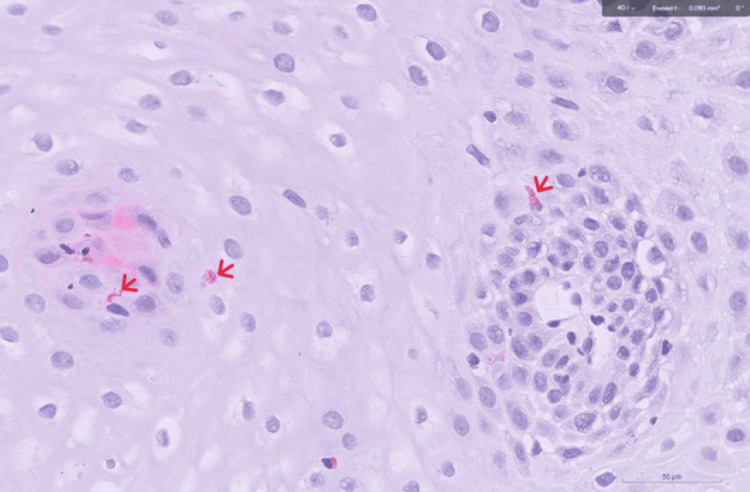
Histopathology of the distal esophagus showing eosinophils (red arrows)

**Figure 5:**
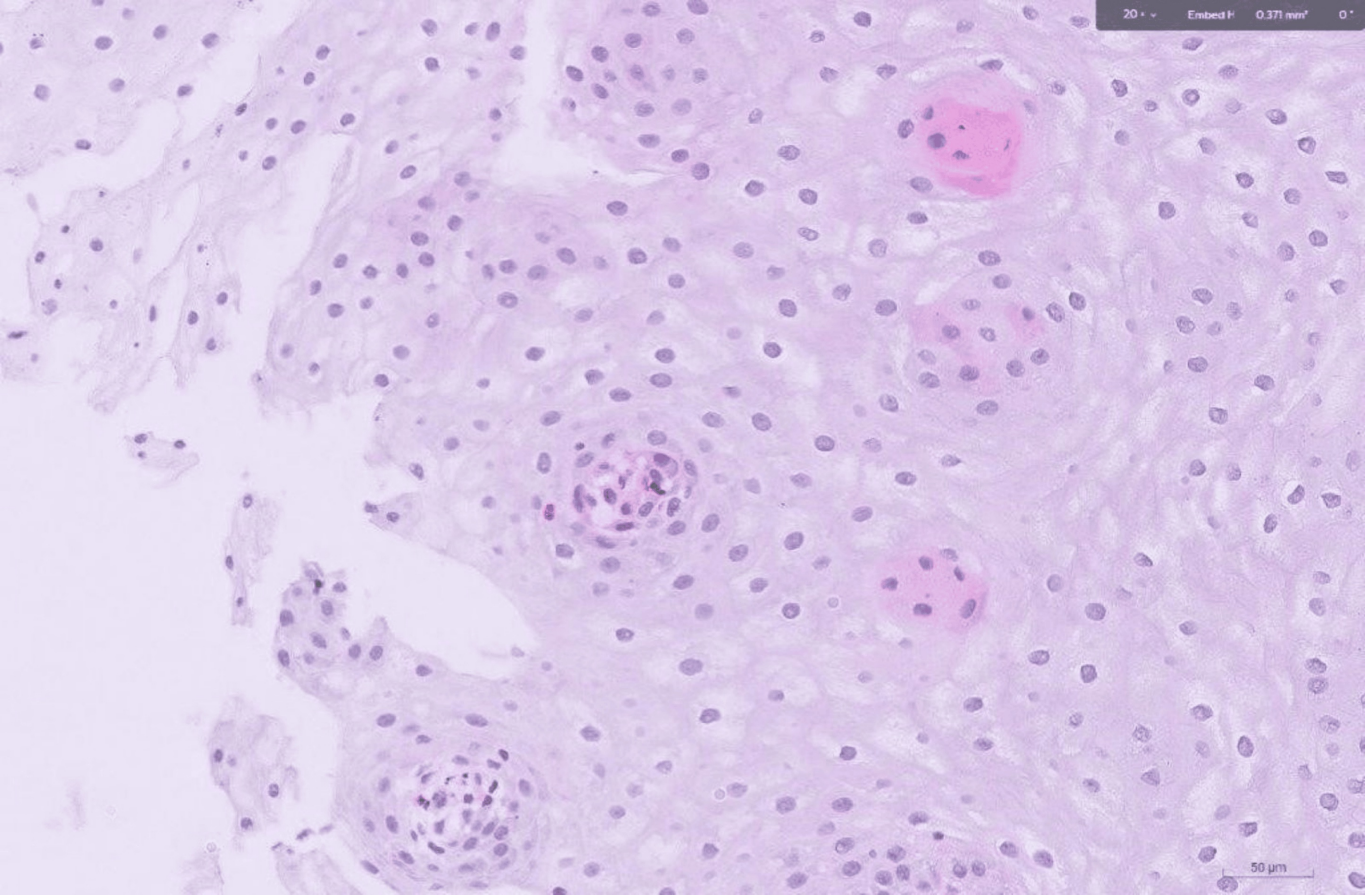
Histopathology of the middle esophagus

**Figure 6:**
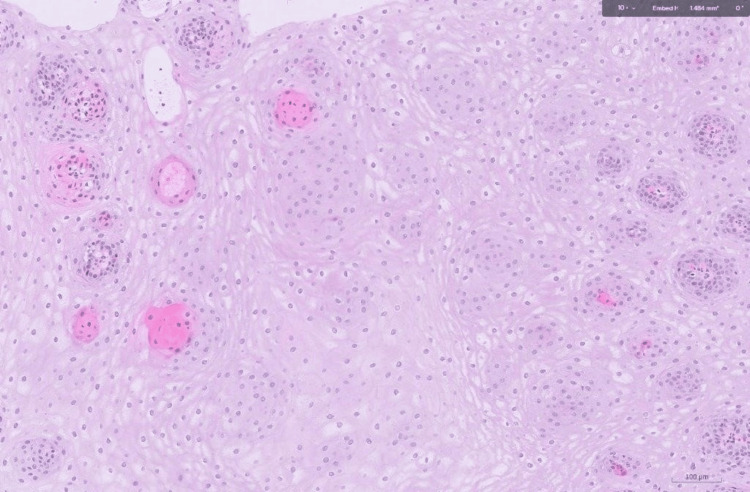
Histopathology of the proximal esophagus

Between then and September 2024, the patient had become asymptomatic on dupilumab with no side effects reported. He underwent two EGD with esophageal dilations tailored by an endoluminal functional lumen imaging probe (EndoFlip) (Figures 2, 3). On his last EGD in June 2024, his (EoE-EREFS) total score was 2; edema: 1, rings: 0, exudates: 0, furrows: 0, and stricture: 1. The pathology report, on the other hand, showed 4 eos/hpf in the distal esophagus (Figure 4) compared to 1 eos/hpf in the biopsies taken from the middle (Figure 5) and proximal esophagus (Figure 6).

## Discussion

Since the discovery of corticosteroids in the 1940s [7], they have been utilized for various purposes. Initially, these compounds were believed to be beneficial only for patients with Addison’s disease. At this present time, the primary clinical applications of steroids are their strong anti-inflammatory and immune-modulating effects, which are used in many systems like GIT, respiratory, skin, etc. However, steroids are also associated with a range of significant side effects that are dose-dependent and affected by the route administered, ranging from simple acne to severe conditions such as Cushing’s syndrome [8].

One of the well-known side effects of corticosteroids is AVN of bone. It is a condition that can range from asymptomatic to debilitating disease. When the blood supply of the acetabulum of the femoral head is disrupted, ischemia may occur. This condition can be triggered by the prolonged use of high-dose steroids. It is believed that glucocorticoids impact lipid metabolism, leading to the creation of lipoprotein globules and fat emboli. These substances can obstruct peripheral blood vessels, ultimately causing bone necrosis due to reduced blood flow [9]. Not only corticosteroids use but also underlying clinical conditions may play a role in developing AVN, such as sickle cell disease, inflammatory bowel disease, SLE, and many others [10]; however, EoE was not reported in the literature to be associated with AVN. Studies that have been conducted before suggested dose, prolonged duration, and route of administration, which is usually parenteral or peroral, are associated with developing AVN [10]. Few cases have been reported in which topical corticosteroids can cause AVN. A case described bilateral AVN of patellae after inhaled corticosteroid therapy was used for the treatment of asthma [6], and another case report showed that inhaled corticosteroid caused AVN in a patient known to have asthma [11]. Moreover, a 10-year-old asthmatic male suffered from inhaled steroid-induced osteonecrosis of the right hip [12].

EoE is a chronic, localized, immune-mediated condition of the esophagus. Clinically, it is marked by esophageal dysfunction, while histopathologically, it is characterized by inflammation predominantly involving eosinophils [13]. To diagnose this condition, more than 15 eos/hpf on light microscopy on a biopsy, with the elimination of secondary causes of esophageal eosinophilia, like other inflammatory intestinal conditions, such as gastroesophageal reflux disease (GERD), inflammatory bowel disease, celiac disease, and extraesophageal eosinophilic gastrointestinal disorders [14].

Treatment of EoE includes a food elimination diet, PPIs, swallowed topical corticosteroids [15], and monoclonal antibodies like dupilumab [16]. 

We are reporting a patient with EoE who developed bilateral AVN of the hips on the prolonged treatment of swallowed fluticasone. This patient required surgical interventions and ended up with bilateral hip replacement. Based on a thorough review of the available medical literature, to the best of our knowledge, this is the first case report for such an entity. Additionally, there are no case reports linking EoE with AVN. The association of systemic use of corticosteroids and AVN is well documented in the literature, but a similar link between topically applied steroids and inhalers is not well established, as mentioned earlier.

It might be challenging to confirm with high certainty the causality between the prolonged use of swallowed fluticasone and AVN in our case, but in our defense, the patient does not have other obvious risk factors at such a young age. Although someone might entertain the thought of EoE increasing the risk for such complications, such a possibility has not been reported or established as far as we know. Our patient received swallowed fluticasone for seven years, which was stopped only when he developed a serious complication, as systemic side effects were less expected. In an attempt to assess the efficacy of the maintenance dose of swallowed topical corticosteroids among patients with EoE, Greuter et al. reviewed 229 patients over five years; the most common side effect observed was esophageal candidiasis (2.7% of the cohort). However, AVN was not reported [17].

## Conclusions

In conclusion, we hereby report the first case of bilateral AVN secondary to prolonged swallowed fluticasone in a patient with EoE. We believe our case report will raise a question about the safety of swallowed topical corticosteroids. Even further, similar case reports will enhance the awareness among health care providers to address such long use of these medications in their practice and hopefully draw the attention of researchers to conduct well-designed studies to explore in depth the association between topical swallowed/inhaled steroids and those rare side effects.
